# A Multi-PTM omics atlas uncovers novel aging regulators in colorectal cancer

**DOI:** 10.1186/s12885-025-15274-7

**Published:** 2025-12-12

**Authors:** Yujie Zhang, Wei Zhang, Tianyuan Li, Erjiao Hao, Jie Du, Min Feng, Feng Zhu, Yong Dai

**Affiliations:** 1 School of Medicine, Anhui University of Science & Technology, Huainan, 232001 China; 2 Department of Clinical Laboratory, Peking University Shenzhen Hospital, Shenzhen, 518036 China; 3 Guangdong Provincial Clinical Research Center for Laboratory Medicine, Guangzhou, 510515 China; 4 The First Hospital of Anhui University of Science and Technology, Huainan, 232001 China; 5 Key Laboratory of Industrial Dust Deep Reduction and Occupational Health and Safety of Anhui Higher Education Institutes, Huainan, 232001 China; 6 Anhui Provincial, Academician Workstation of Anhui University of Science & Technology for Autoimmune Disease Diagnostic Technology, Huainan, 232001 China

**Keywords:** Colorectal cancer (CRC), Aging, Post translational modifications (PTMs), Multiomics, Phosphorylation, Malonylation, Ubiquitination

## Abstract

**Background:**

Aging is a key driver of colorectal cancer (CRC) progression, yet the post-translational modification (PTM) landscape associated with aging in CRC remains largely uncharacterized. In particular, the coordinated influence of multiple PTMs—such as phosphorylation, ubiquitination, and malonylation—on aging-related pathways has not been systematically explored.

**Methods:**

In this study, we established a CRC-specific multiomics framework by profiling phosphorylation, malonylation, and ubiquitination in matched tumor and adjacent normal tissues (n = 8 pairs). The differentially modified proteins were subjected to functional enrichment, protein–protein interaction (PPI) network construction, structural mapping, and aging pathway annotation. Key regulatory axes were reconstructed through integration of GO, KEGG, and literature-based evidence.

**Results:**

Aging-related PTMs were extensively dysregulated in CRC, with 162 ubiquitination sites, 64 phosphorylation sites, and 68 malonylation sites altered. LMNB1 has emerged as a multi-PTM protein, indicating coordinated control of the nuclear structure during senescence. PPI network analysis highlighted CDK1, SOD2, and MAPK1 as potential hub PTM-regulated nodes involved in the aging program of CRC. An integrated signaling model further demonstrated how PTM-mediated suppression of the EGFR–RAS axis, along with activation of the p38 and p53 pathways, collectively contributes to shaping the aging phenotype in CRC.

**Conclusion:**

This study presents the first integrative network map of aging regulation in CRC based on multiple PTMs. Notably, hub proteins such as LMNB1 have emerged as key regulatory targets. These findings provide a theoretical foundation for the development of aging–cancer axis–related biomarkers and therapeutic strategies for CRC.

## Introduction

Aging is a major risk factor for the initiation and progression of colorectal cancer (CRC), profoundly shaping the tumour immune microenvironment, therapeutic response, and clinical outcome [1]. Unlike discrete oncogenic events, aging represents a global biological process that integrates multiple layers of cellular dysfunction, ranging from genomic instability and epigenetic drift to metabolic imbalance and immune senescence [2, 3]. These intertwined alterations collectively reshape the tumor microenvironment, thereby driving malignant evolution and determining clinical outcomes [4]. However, despite its fundamental role, the molecular basis through which aging promotes CRC development remains incompletely elucidated.

Traditional CRC studies often focus on isolated hallmarks such as DNA damage or metabolic rewiring [3, 5], yet these individual processes only capture fragments of a broader system-level phenomenon. Aging provides a unifying framework to understand how diverse molecular perturbations converge to facilitate tumorigenesis [6]. By investigating aging as an integrative biological axis, rather than as separate mechanisms, we can delineate the coordinated regulatory networks that underlie CRC initiation or progression.

Among the multifaceted molecular regulators of aging, post-translational modifications (PTMs), including phosphorylation, ubiquitination, and malonylation—represent key modulators of protein activity and signaling dynamics [7, 8]. Their reversible, enzyme-dependent nature enables precise control of stress responses and senescence programs, while their dysregulation often marks early pathological transitions [9]. Yet, systematic characterization of PTM dynamics in CRC-associated aging remains largely unexplored.

In this study, we presented a multiomics research framework to comprehensively dissect the aging-associated molecular landscape in CRC. By integrating multiple layers of proteomic data, we aim to uncover the PTM-driven signaling architecture that links aging to tumor biology, offering new insights into potential biomarkers and therapeutic targets for aging-related colorectal cancer.

## Materials and methods

### Data sources

The proteomics and PTMs datasets used in this study were derived from an in-house CRC cohort established by our laboratory and included phosphorylation (nCRC = 8, nNC = 8) and ubiquitination (nCRC = 8, nNC = 8) data [10, 11]. Malonylation proteomics data (nCRC = 8, nNC = 8) were generated for the first time in this study via affinity enrichment combined with high-resolution mass spectrometry (the detailed methods are described below). The aging-related gene set was obtained from the HPA database [12]. Protein structure and sequence information were retrieved from the UniProt database, which served as the foundation for subsequent modification site localization and functional annotation analyses. We performed additional analyses with an emphasis on aging-related features of these data.

### Patient information

Patients who are diagnosed with primary CRC at Shenzhen People’s Hospital and who underwent surgical resection without receiving preoperative radiotherapy or chemotherapy were enrolled in this study. This study used paired cancerous and adjacent normal tissue samples from the same CRC patients (n = 8), focusing on molecular-level senescence-associated features rather than chronological age, thereby eliminating age-related bias. Individuals with hereditary forms of CRC were excluded. Each case was evaluated and graded by gastroenterologists on the basis of pathological assessment. Clinical data, histopathological characteristics, and treatment-related information were retrieved from the patients’ electronic medical records. All participants were fully informed about the study and voluntarily signed written informed consent forms. The study protocol was approved by the Ethics Committee of Shenzhen People’s Hospital (Approval No. LL-KY-2019213).

### Detection of lysine malonylation

Lysine malonylation profiling was performed on CRC and matched normal tissues (nCRC = 8, npara-CRC = 8) via a 4D label-free proteomics approach. Following tissue lysis, protein quantification, and tryptic digestion, malonylated peptides were enriched via immunoaffinity purification via the use of anti-malonyllysine antibodies. The enriched peptides were separated by nanoflow liquid chromatography and analyzed on the Bruker timsTOF Pro mass spectrometry platform, employing four-dimensional separation on the basis of retention time, m/z, charge state, and ion mobility to increase detection sensitivity and depth. The raw mass spectrometry data were processed with MaxQuant software by searching against the UniProt human database, with lysine malonylation (+ 100.0160 Da) set as a variable modification. The false discovery rate (FDR) was controlled below 1%. High-confidence modification sites were obtained for subsequent quantitative analysis and bioinformatic investigations.

### Database searching

The secondary MS data were analyzed using the MaxQuant search engine (version 1.6.6.0) with the Andromeda algorithm. Spectra were searched against the Homo sapiens UniProt database (taxon ID 9606; 20,366 sequences) combined with the common contaminant database provided by MaxQuant. A reverse decoy database was included to estimate the false discovery rate (FDR) arising from random matches.

All search parameters were configured as follows: the FDR thresholds for both protein and peptide-spectrum matches (PSMs) were set at 1%. The enzyme specificity was set to trypsin/P, allowing up to four missed cleavages. The minimum peptide length was seven amino acids, and the maximum number of variable modifications per peptide was five. The mass tolerance for precursor ions in the first search was 20 ppm, and 0.02 Da for fragmentions. Carbamidomethylation of cysteine (C) was specified as a fixed modification, while acetylation (protein N-terminus), oxidation of methionine (M), and diglycine modification on lysine (K) were defined as variable modifications [11, 13].

### Functional enrichment analysis

Functional annotation and protein–protein interaction (PPI) network analysis of the differentially expressed proteins were performed via the Metascape platform (https://metascape.org/gp/index.html). Metascape is an integrated online resource that combines multiple bioinformatics databases to facilitate functional annotation, pathway enrichment, and PPI network visualization for differentially expressed genes or proteins [14]. The list of filtered differentially expressed proteins was uploaded to Metascape, with a significance threshold set at p < 0.05 after Benjamini–Hochberg correction. Enrichment was evaluated using the hypergeometric test. Significant pathways were further clustered using the built-in MCODE algorithm to generate functional module network maps.

### Construction of the PPI network and identification of hub proteins

The PPI network was constructed using the STRING database (https://string-db.org), with a confidence score threshold above 0.7. After the network data were exported, visualization and topological analyses were performed using Cytoscape software. Hub proteins were identified on the basis of degree centrality and betweenness centrality metrics. The network submodules were further analyzed via import into Metascape’s MCODE algorithm for secondary functional enrichment analysis.

### Protein domain analysis

The amino acid sequences and three-dimensional structures of the target proteins were obtained from the UniProt database (https://www.uniprot.org/) [15]. To further analyze the spatial distribution of the modification sites, the crystal structures of the target proteins were visualized using PyMOL molecular visualization software (PyMOL v3.1.3.1).

### Localization and annotation of PTMs sites

The UniPort website was used to identify protein linear structures (https://www.uniprot.org/). The specific positions of each modification were mapped onto the protein sequences and three-dimensional structures, and then color-coded and visualized using PyMOL software [16].

### Pathway network identification

To systematically elucidate the potential aging regulatory network in CRC, all significantly differentially modified proteins identified in CRC tissues were first integrated. The functions of these genes in aging-related biological processes—such as cellular senescence, DNA damage repair, epigenetic regulation, chromatin remodeling, and cell cycle control—were subsequently categorized based on annotations from the Gene Ontology (GO) and Kyoto Encyclopedia of Genes and Genomes (KEGG) databases, supplemented by relevant reviews and experimental studies retrieved from PubMed. Furthermore, leveraging reported protein regulatory relationships and interactions among biological processes, a putative aging regulatory pathway network was constructed to depict the key mechanistic landscape by which differentially modified proteins mediate aging regulation in CRC.

#### Statistical analysis

Group comparisons were performed using independent t-tests, with statistical significance defined by *P* values as follows: *P < 0.05; **P < 0.01; ***P < 0.001; ****P < 0.0001.

## Results

### Significant differential expression of multiple PTMs in CRC tissues

PTMs play crucial regulatory roles in cellular senescence and tumor progression [17], however, the comprehensive modification landscape under aging conditions in CRC remains unexplored. Phosphorylation, malonylation, and ubiquitination are among the most common and biologically significant PTMs and are extensively involved in aging- and cancer-related signal transduction, metabolic reprogramming, and cell cycle regulation [18, 19]. These PTMs regulate CRC initiation, progression, and metastasis by modulating protein function, stability, and interactions during aging.

**Fig. 1:**
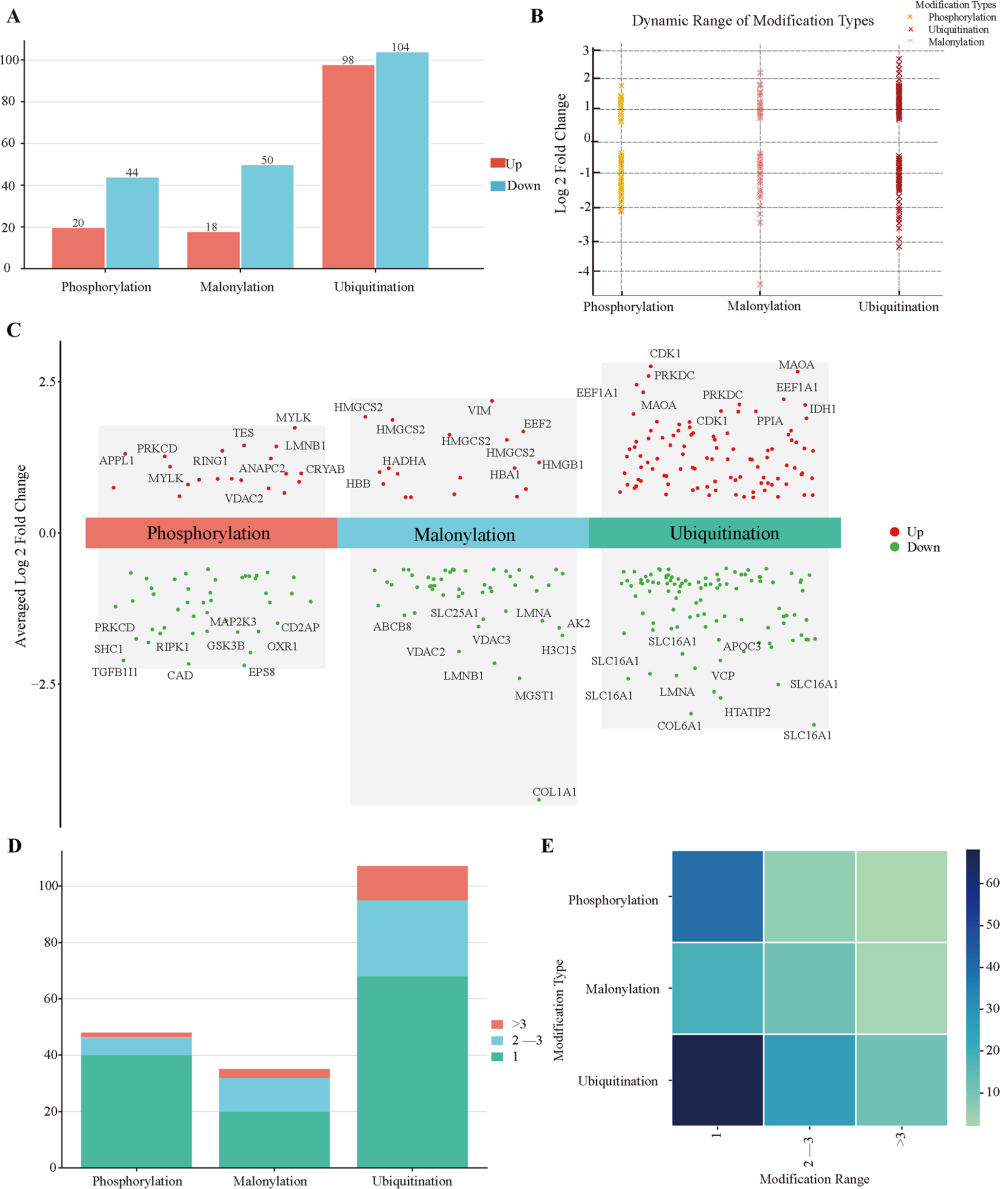
Differential expression patterns of three types of aging-related protein PTMs in CRC tissues. **A** Bar plots showing the number of upregulated and downregulated sites for phosphorylation, ubiquitination, and malonylation in CRC and normal tissues (*n* = 8 pairs). The color intensity indicates the magnitude of change, with red indicating upregulation and blue indicating down-regulation. **B** Scatter plots illustrating the distribution of differential sites for the three PTM types across the tumor and normal tissues. A positive fold change (greater than 0) indicates a positive correlation (upregulation in tumor tissue), while a negative fold change (less than 0) indicates a negative correlation (downregulation in tumor tissue). The further the value is from 0, the more statistically significant the change is. **C** Heatmap displaying the average log₂ fold change (log₂FC) in modified protein expression across different PTM types. The color intensity indicates the magnitude of change, with red indicating upregulation and green indicating downregulation. *p < 0.05. **D** Bar plot of differential modification sites across all PTM types. Green represents proteins with one modified site, blue represents proteins with 2–3 modified sites, and red represents proteins with more than three modified sites. **E** Heatmap of differential modification sites. The darker the color, the greater the number of proteins

Accordingly, this study analyzed the expression patterns of phosphorylation, malonylation, and ubiquitination in CRC tissues and their matched adjacent normal controls. The results revealed that, at the ubiquitination level, 98 modification sites were up-regulated and 104 sites were down-regulated; at the phosphorylation level, 20 sites were up-regulated and 44 were down-regulated; and at the malonylation level, 18 sites were up-regulated and 50 were down-regulated **(**Fig. 1A**)**. The fold changes of these three PTM types in tumor versus adjacent tissues exhibited dynamic variations, with maximum differences reaching 2.8-fold **(**Fig. 1B**)**.

Volcano plots further demonstrated significant up- or down-regulation of PTMs on multiple critical tumor proteins, such as MYLK, CDK1, and PRKDC (Fig. 1C). These proteins are subject to multiple PTMs, underscoring their central roles in CRC pathogenesis [20–22]. Moreover, several proteins exhibited simultaneous upregulation at multiple modification sites, exemplified by ERBB2 (Fig. 1D, E), suggesting a highly active modification environment and reflecting global epigenetic dysregulation in CRC.

Collectively, these findings indicate extensive heterogeneity and widespread remodeling of PTMs during CRC-associated aging, implying that multiple modifications may synergistically regulate key protein functions and jointly drive aberrant activation of tumor-associated aging programs.

### Multiple PTMs of the nuclear lamina core protein LMNB1 in CRC

**Fig. 2:**
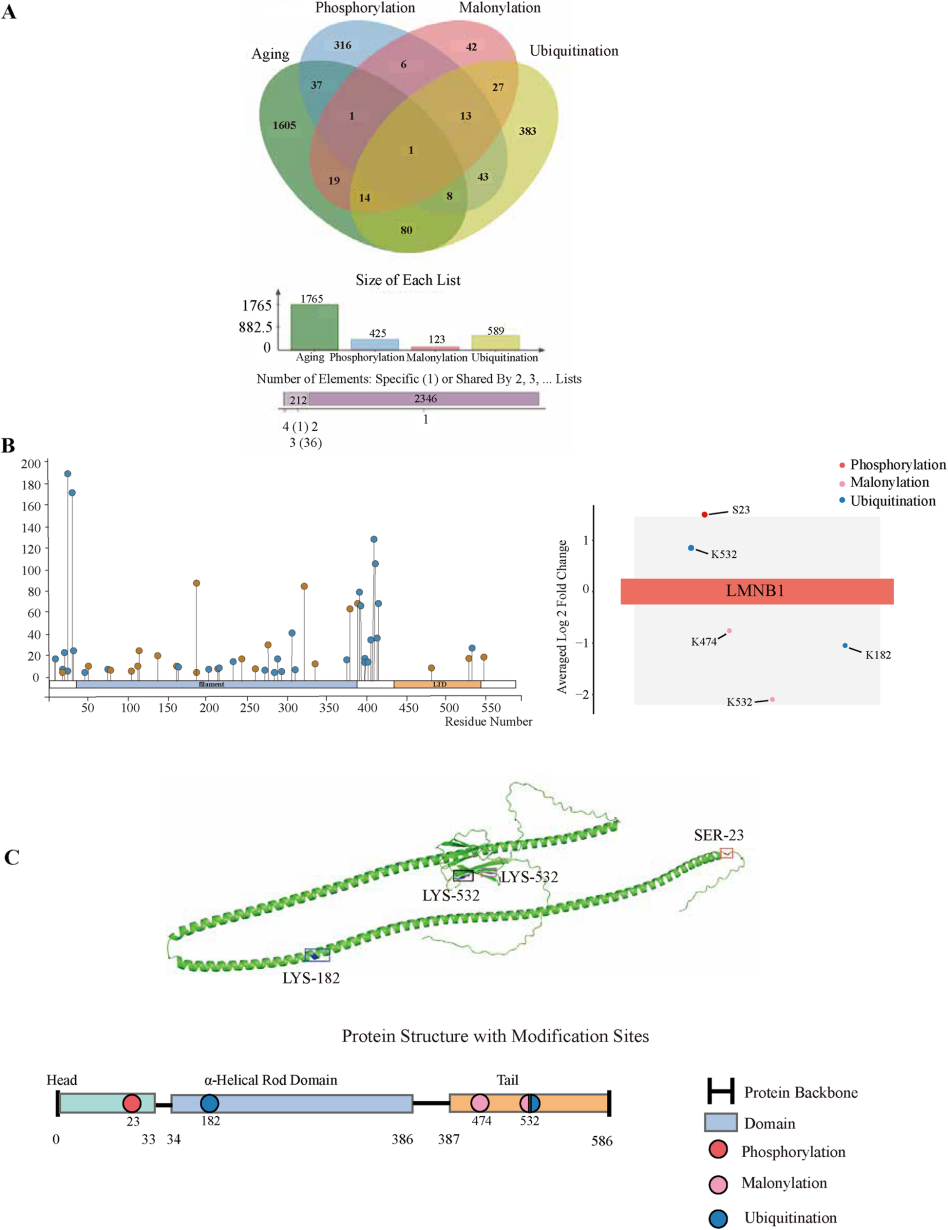
LMNB1 exhibits three types of modification level changes in CRC vs NC. **A** Venn diagram showing the intersection of differentially phosphorylated, malonylated, and ubiquitinated proteins in CRC tissues. Highlighting the overlap and unique modification sites for each type of PTM across CRC tissues. **B** Illustrating the differential expression of LMNB1 at various PTMs sites. The x-axis of the left plot represents the LMNB1 protein sites, and the y-axis represents the number of references. The X-axis of the right plot represents the different LMNB1 modification sites, and the Y-axis shows the number of references for each modification site. The log₂ fold change (Y-axis) indicates the magnitude of upregulation or downregulation at each site, with significant changes marked accordingly. Positive fold change values indicate upregulation in CRC tissues, while negative values show downregulation. *p < 0.05. **C** Three-dimensional structure diagram of LMNB1 protein. The red represents phosphorylation, blue represents ubiquitination, and pink represents malonylation. Protein structure visualization was performed using PyMOL (v3.1.3.1) based on UniProt entry P20700

Proteins subjected to multiple PTMs often act as integrative nodes within complex signaling networks, especially in aging and tumor biology. Based on this rationale, we analyzed proteins exhibiting concurrent changes in phosphorylation, malonylation, and ubiquitination. Our results **(**Fig. 2A**)** revealed that LMNB1 undergoes multiple PTMs, including upregulated phosphorylation at Ser23, increased ubiquitination at Lys532, decreased ubiquitination at Lys182, and decreased malonylation at Lys474 and Lys532 **(**Fig. 2B**)**.

As a core component of the nuclear lamina, LMNB1 plays critical roles in maintaining nuclear structural integrity, regulating chromatin organization, and mediating DNA repair [23–25]. Previous studies have shown that LMNB1 expression and modification are closely linked to cellular senescence; aberrant upregulation or dysregulation of its PTMs can lead to chromatin decondensation, nuclear envelope disruption, and cell cycle abnormalities [26]. The observed coexistence of multiple PTMs on LMNB1 in this study likely reflects the convergence of distinct signaling pathways—such as the stress response, metabolic reprogramming, and protein degradation—at this pivotal node, triggering structural and functional remodeling.

We further evaluated the academic attention (i.e., functional awareness) of LMNB1’s modification sites (Fig. 2B), noting that phosphorylation at Ser23 is relatively well characterized. Reports indicate that in neurodegenerative diseases such as Alzheimer’s disease, phosphorylation at LMNB1 Ser23 leads to rapid nuclear dispersal, serving as an early trigger of cellular senescence and apoptosis [27].

Structural mapping of LMNB1 revealed a non-random spatial distribution of the three PTM types—phosphorylation, malonylation, and ubiquitination—across distinct structural domains (Fig. 2C). Specifically, phosphorylation occurs in the head domain (Ser23), suggesting a role in regulating nuclear lamina assembly and protein conformation stability; ubiquitination localizes to the central α-helical rod domain (Lys182) and the tail domain (Lys532), potentially influencing interactions with chromatin or the nuclear membrane; and malonylation sites cluster in the tail region (Lys474, Lys532), implying involvement in LMNB1 degradation, trafficking, or functional modulation. This domain-specific multi-modification pattern suggests that LMNB1 may be coordinately regulated by complex PTM crosstalk in CRC, impacting nuclear architecture integrity, chromatin organization, and the transmission of senescence signals.

Notably, the enrichment of multiple modifications in the tail domain highlights this region as a regulatory hub for LMNB1 stability and dynamic remodeling, providing a structural basis for future functional studies targeting multi-site PTM modulation of this protein.

## Multilayered functional stratification of PTM-regulated aging pathways in CRC

**Fig. 3:**
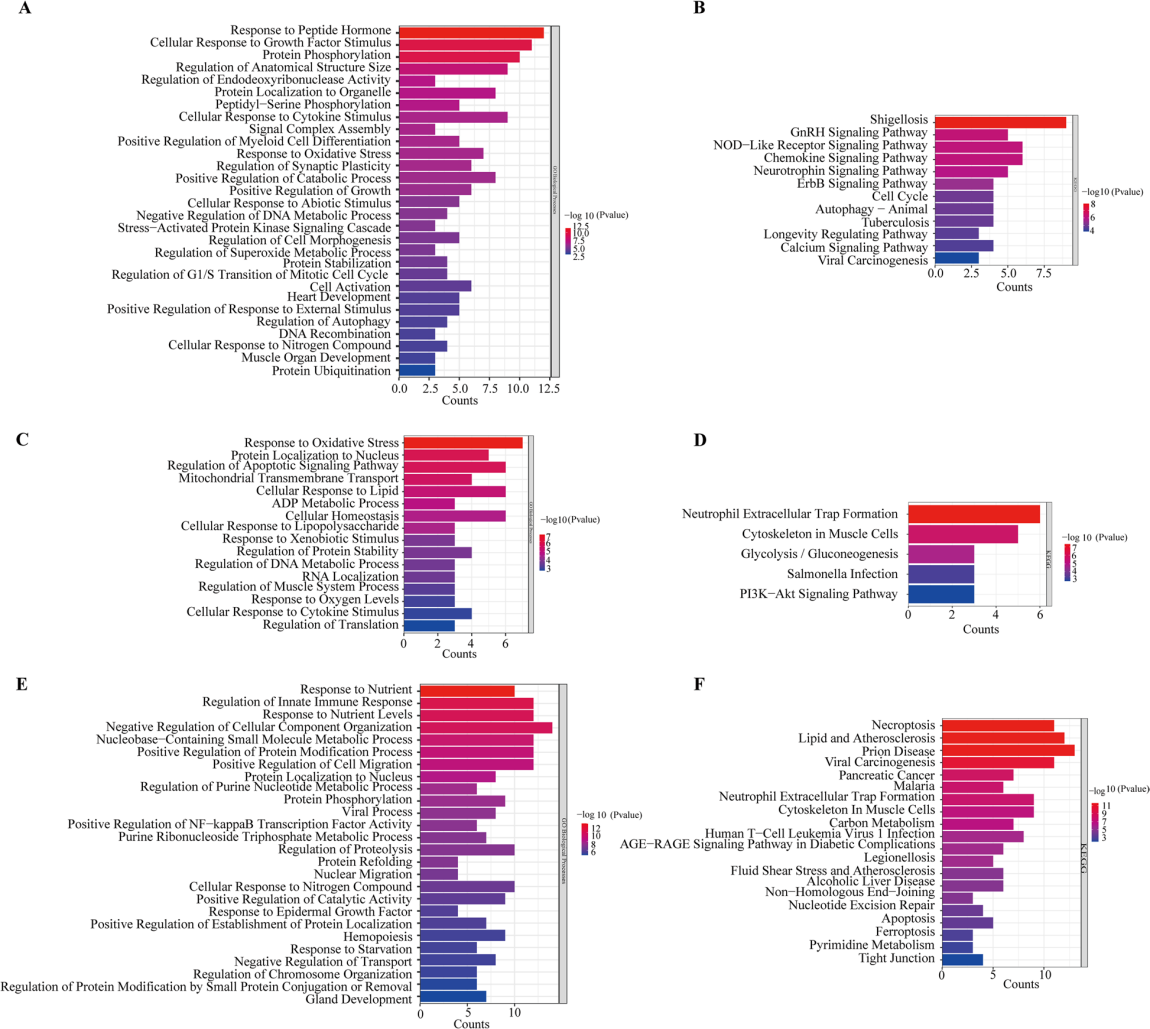
Functional annotation of three types of differential modificated proteins. **A** GO annotation diagram of the differentially phosphorylated proteins. **B** KEGG annotation diagram of the differentially phosphorylated proteins. **C** GO annotation diagram of differentially malonylated proteins. **D** KEGG annotation diagram of differentially malonylated proteins. **E** GO annotation diagram of differential ubiquitination proteins. **F** KEGG annotation diagram of differential ubiquitination proteins. All GO plots represent Biological Process (BP) terms associated with differentially modified proteins. The x-axis represents the number of proteins, the y-axis represents the pathways associated with differentially modified proteins, and the color represents the significance (the stronger the red, the more reliable the result)

To clarify which aging-related pathways are regulated primarily by phosphorylation, malonylation, and ubiquitination, we performed enrichment analyses on differentially modified proteins in CRC. The results revealed that proteins with differential phosphorylation were enriched mainly in classical aging-related pathways such as the MAPK signaling pathway, cell cycle regulation, and p53 signaling **(**Fig. 3A–B**)**. The differentially expressed malonylated proteins were predominantly associated with the tricarboxylic acid (TCA) cycle, lipid metabolism, and mitochondrial function, indicating their involvement in metabolic homeostasis regulation **(**Fig. 3C–D**)**. Moreover, the differentially ubiquitinated proteins were significantly linked to protein degradation, DNA repair, and autophagy pathways, reflecting their central roles in protein quality control and genomic stability **(**Fig. 3E–F**)**. These findings support a multilayered regulatory model whereby distinct types of PTMs contribute to the initiation, maintenance, and adaptive feedback regulation of aging programs in CRC, collectively shaping tumor cell responses to senescence signals.

### Spatial and functional coordination of PTMs in CRC-associated senescence

**Fig. 4:**
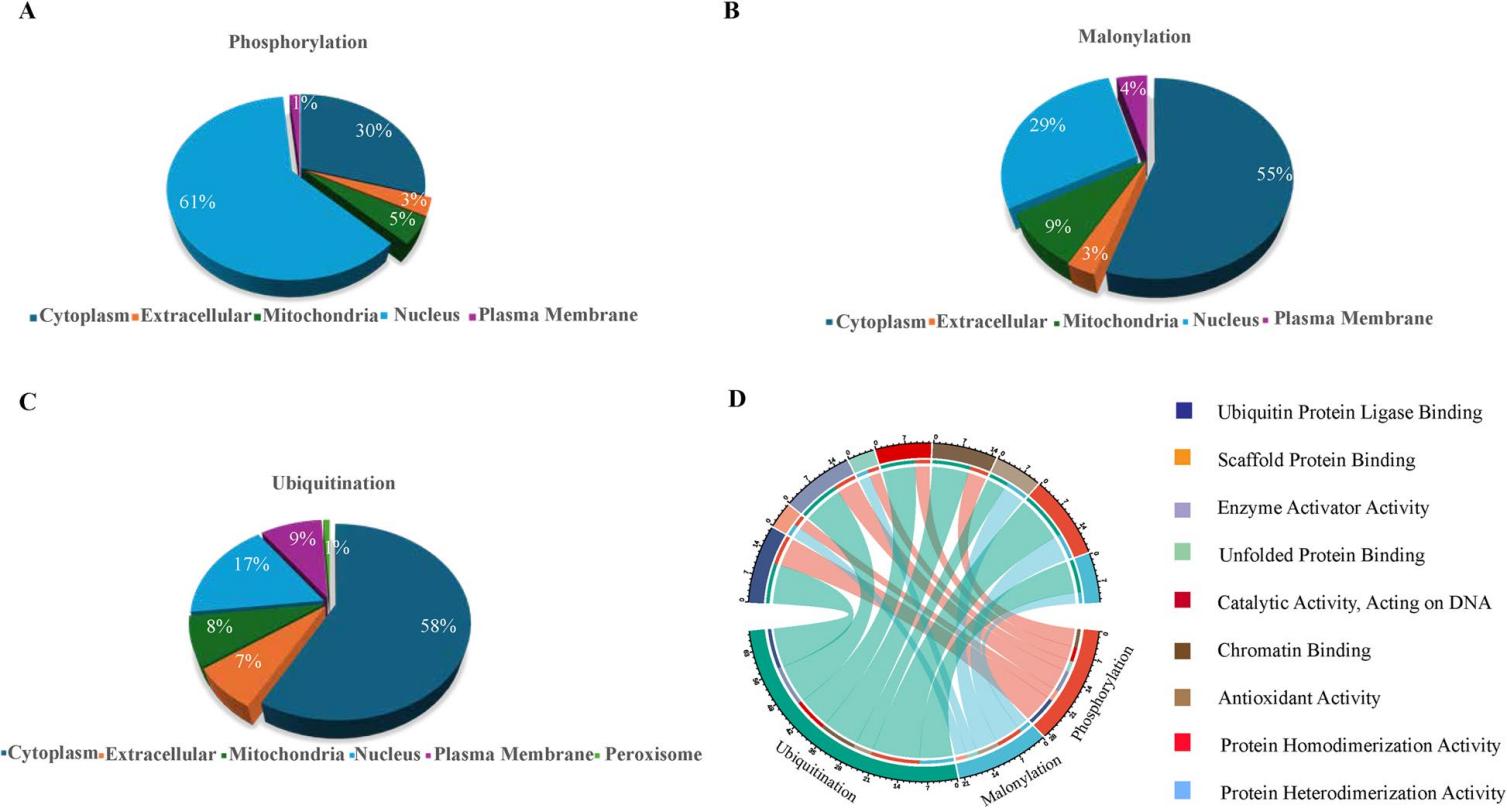
Subcellular localization analysis and molecular function analysis of three differentially modified proteins. **A** Pie chart showing the subcellular localization of differentially phosphorylated proteins. **B** Pie chart showing the subcellular localization of proteins modified by differential malonylation. **C** Pie chart showing the subcellular localization of differentially ubiquitinated proteins. **D** Chord diagram of functional pathways and modification types. Chord diagram illustrating the overlap in GO molecular functions among phosphorylation, malonylation, and ubiquitination datasets

Furthermore, we investigated whether proteins modified by different PTMs exhibit specific subcellular localizations. Cellular component annotation revealed that phosphorylated proteins were predominantly cytoplasmic (61%) **(**Fig. 4A**)**, and malonylated proteins were significantly enriched in the mitochondria and cytoplasm (58% and 29%, respectively) **(**Fig. 4B**)**, whereas ubiquitinated proteins were distributed not only in the cytoplasm but also in the nucleus, plasma membrane, and peroxisomes (17%, 9%, and 7%, respectively) **(**Fig. 4C**)**. These results indicate that different PTM-modified proteins display distinct subcellular distribution patterns, supporting their potential cooperation within specific cellular microenvironments to mediate aging-related processes.

To further explore the functional diversity of the differentially modified proteins, we performed GO molecular function enrichment analysis and visualized the functional convergence across phosphorylation, malonylation, and ubiquitination using a chord diagram (Fig. 4D). The results revealed extensive overlaps in several key functions, including catalytic activity on DNA, antioxidant activity, chromatin binding, and scaffold protein binding. Phosphorylated proteins are particularly associated with enzyme activator activity and protein dimerization functions, whereas ubiquitinated proteins are enriched in unfolded protein binding and ubiquitin protein ligase binding. Notably, Malonylated proteins are linked to chromatin-associated functions and scaffold protein binding. These findings support a model in which different PTMs converge on overlapping yet distinct functional roles, potentially contributing to the layered regulation of aging processes in CRC.

### Differential PTMs define hubs and functional modules that interaction in CRC-related senescence

**Fig. 5:**
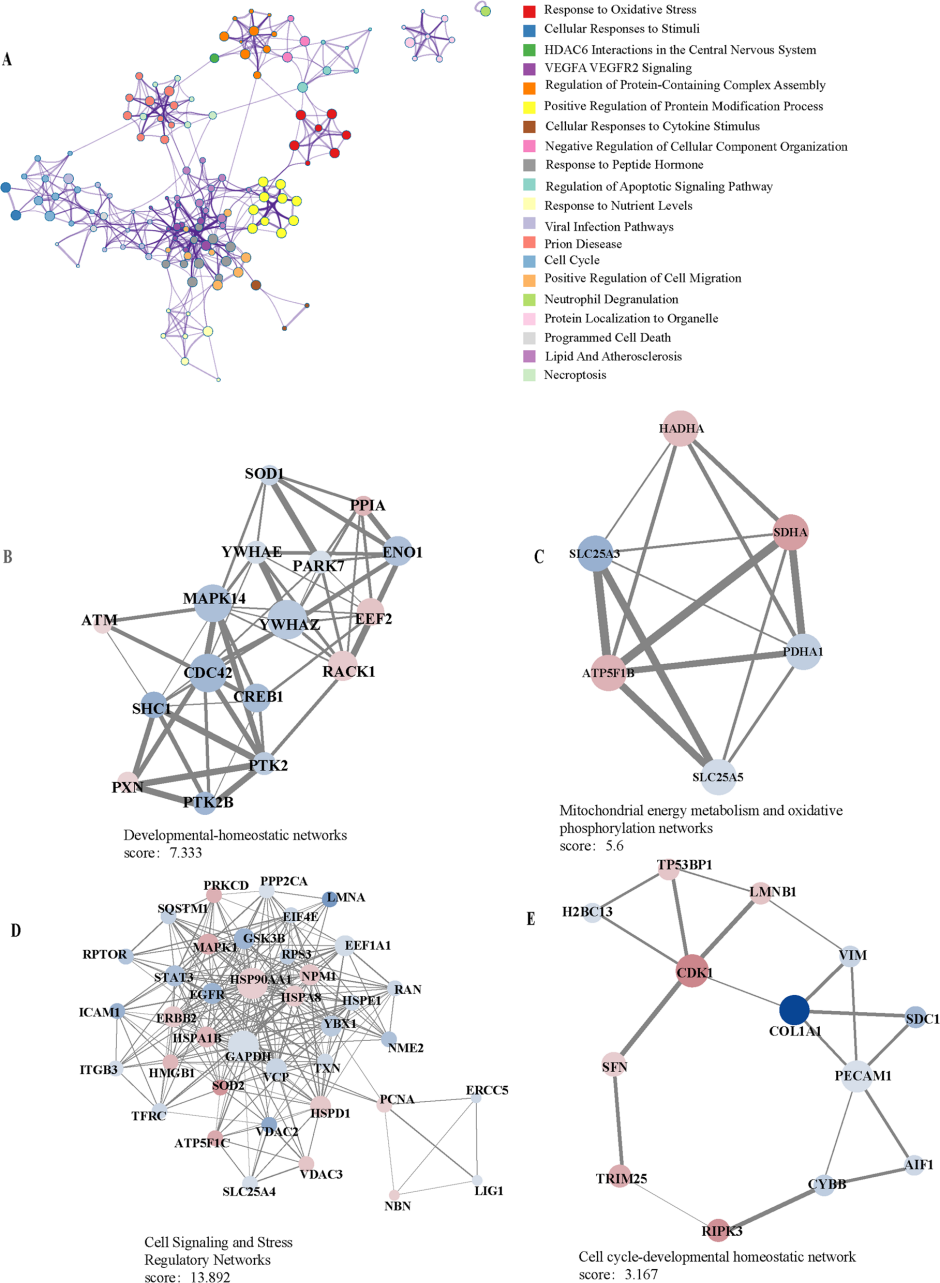
Regulatory interaction network of differentially modified aging-related proteins and functional module. **A** Global PPI network showing interactions among differentially modified proteins. This network highlights proteins involved in cellular senescence and aging-related pathways. It shows how these proteins interact with each other to regulate key biological processes in CRC. **B**-**E** Subnetwork analysis showing functional modules related to mitochondrial metabolism (**B**), oxidative phosphorylation (**C**), signal transduction and stress response (**D**), and cell cycle regulation (**E**)

To elucidate the regulatory networks of phosphorylation, malonylation, and ubiquitination in CRC, we subsequently constructed PPI networks for each PTM class and identified functionally significant modules using the MCODE algorithm. The results demonstrated extensive interactions among these differentially modified proteins, which participate in pathways related to the oxidative stress response and cell cycle regulation **(**Fig. 5A**)**. Subnetwork functions were associated with development and homeostasis maintenance, mitochondrial metabolism and oxidative phosphorylation, signal transduction and stress response, and the cycle and homeostasis modules **(**Fig. 5B–E**)**.

### PTM-mediated remodeling of aging regulatory pathways in CRC

**Fig. 6:**
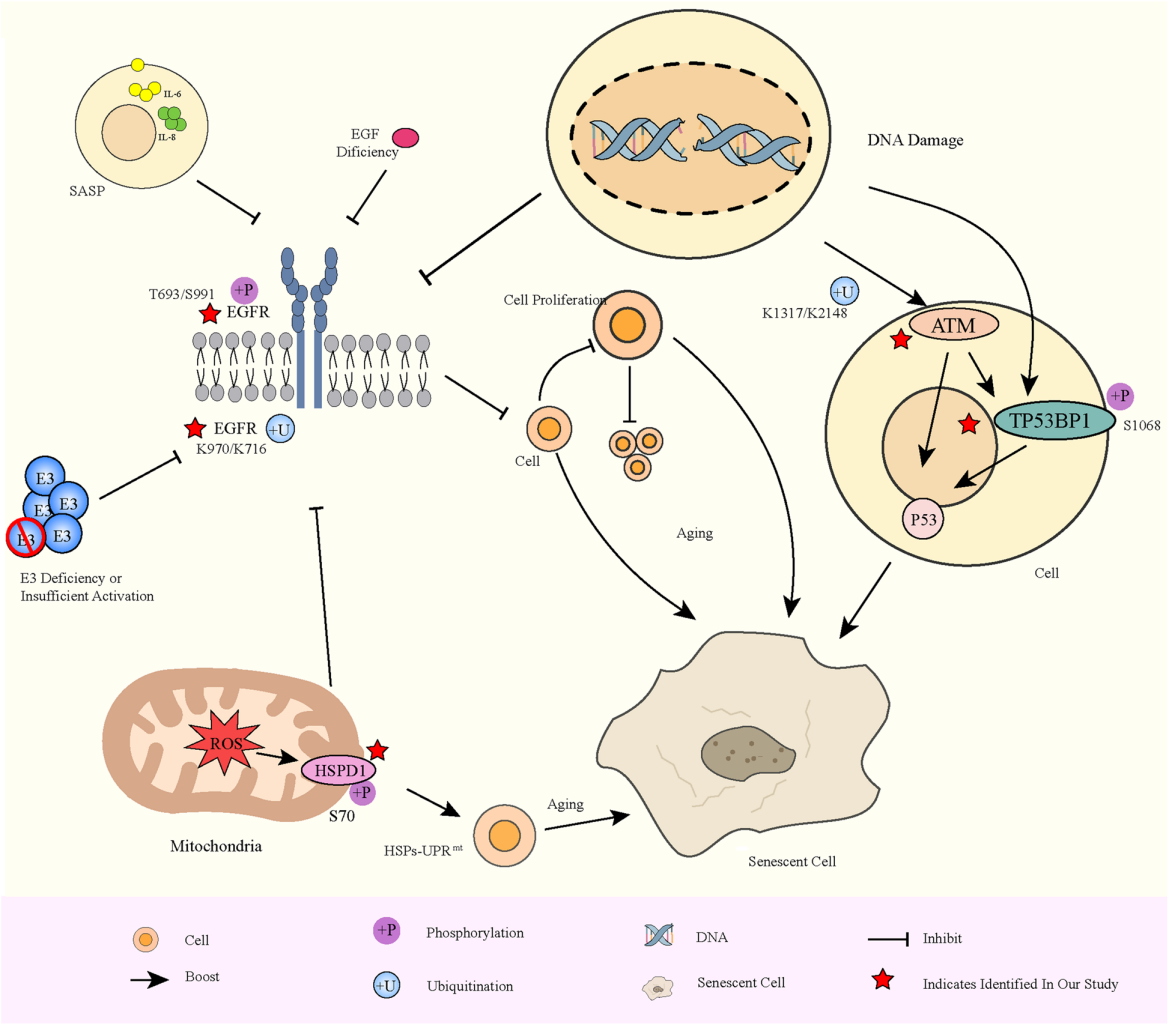
Translation-mediated coordination and regulation of the mechanism model of aging in CRC cells. The schematic diagram integrates the key signaling pathways and core protein nodes involved in PTM regulation, including EGFR signaling inhibition, the ATM-TP53BP1-p21 axis activation, and the HSPD1-mediated mitochondrial stress response. The diagram integrates three major signaling axes: (1) Suppression of the EGFR-RAS-MAPK pathway due to reduced phosphorylation and ubiquitination at specific EGFR residues; (2) Activation of the ATM–TP53BP1-p21 axis via ATM ubiquitination and TP53BP1 phosphorylation; (3) HSPD1 phosphorylation-mediated mitochondrial unfolded protein response (mtUPR). Newly identified modification sites are marked with red stars. Arrows indicate activation, and blunt lines indicate inhibition

To systematically elucidate the potential mechanisms by which differential PTMs regulate aging in CRC, we integrated all significantly differentially modified proteins and performed functional annotation and classification on the basis of GO and KEGG databases, and literature mining. Leveraging known protein regulatory interactions and biological process crosstalk, we constructed a putative aging regulatory pathway network to depict the roles of PTMs in mediating aging processes in CRC **(**Fig. 6**)**.

Our study revealed decreased of phosphorylation at the Epidermal Growth Factor Receptor (EGFR) residues T693 and S991, along with decreased ubiquitination at the K970 and K716 sites. The ubiquitination sites K970 and K716 are novel findings in our research. Previous studies have shown that phosphorylation at T693 and S991 facilitates specific recognition and binding by the F-box and WD repeat domain-containing 7 (FBXW7) E3 ubiquitin ligase, which catalyzes the polyubiquitination of EGFR. This ubiquitination targets EGFR for degradation via the 26S proteasome, limiting its membrane accumulation and signal duration, thereby attenuating EGFR signaling [28–30]. Insufficient EGFR activation weakens downstream signaling through the RAS-RAF-MEK-ERK pathway, inhibiting cellular proliferation and differentiation and promoting entry into senescence [31].

Combining our findings with those of previous reports, the observed reduction in phosphorylation at T693 and S991, accompanied by decreased ubiquitination at K970 and K716, suggests a potential disruption of the canonical EGFR degradation pathway in CRC. This disruption may result in sustained EGFR membrane localization and prolonged signaling, diverging from the expected degradation mechanism mediated by FBXW7 [30]. Alternatively, the attenuated phosphorylation at these key residues may reflect a broader signaling downregulation of EGFR in senescent CRC cells, contributing to reduced RAS-MAPK activity and reinforcing the senescence phenotype.

The novel identification of ubiquitination at K970 and K716 further extends the current understanding of EGFR regulation and indicates the existence of non-canonical ubiquitination sites potentially involved in modulating its stability and function. These results highlight the complex interplay between phosphorylation and ubiquitination in controlling EGFR fate and suggest that altered PTM patterns may underlie CRC-specific senescence regulatory mechanisms.

We also observed upregulated ubiquitination at Ataxia Telangiectasia Mutated (ATM) K1317 and K2148, increased phosphorylation at Tumor Protein p53 Binding Protein 1 (TP53BP1) S1068, and increased phosphorylation upregulation at Heat Shock Protein Family D Member 1 (HSPD1) S70. We identified novel ubiquitination of ATM (K1317, K2148) and phosphorylation of HSPD1 (S70), whereas the phosphorylation of TP53BP1 (S1068) was consistent with previous reports [32].Previous studies have indicated that TP53BP1 functions downstream of ATM signaling, coordinating DNA damage repair and the p53-dependent p21 pathway to induce cell cycle arrest and senescence [33, 34]. These findings suggest that, following DNA double-strand breaks, ATM is rapidly activated via ubiquitination, subsequently phosphorylating multiple downstream targets including TP53BP1. TP53BP1 accumulates at damage sites to recruit repair factors and regulate chromatin remodeling, while cooperating with the p53 pathway to activate p21 expression, causing cell cycle arrest and driving senescence.

Additionally, phosphorylation at HSPD1 S70 was found to be upregulated, and the S70 site is a newly discovered site under investigation. Research has shown that under increased reactive oxygen species (ROS) accumulation, the expression of the mitochondrial chaperone HSPD1 is elevated to enhance the mitochondrial unfolded protein response (mtUPR). Early activation of the mtUPR may exert protective effects during senescence, but excessive or dysregulated activation can accelerate aging progression [35]. Taken together, these findings suggest that phosphorylation at HSPD1 S70 promotes mtUPR overexpression, thereby impacting mitochondrial function and facilitating cellular senescence.

## Discussion

**Fig. 7:**
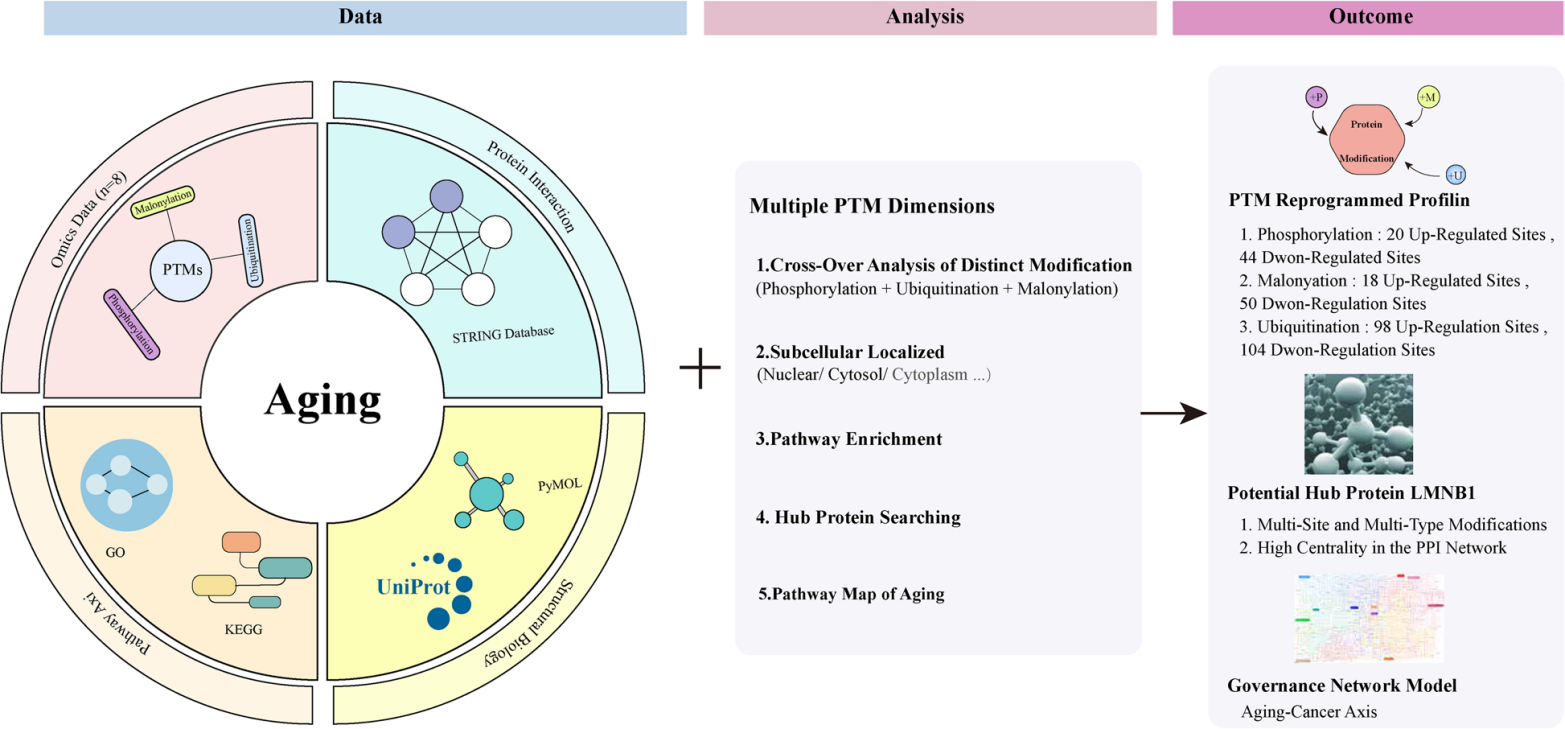
Integrative analysis workflow and summary of key findings. The figure summarizes the experimental design, data integration strategy, main analysis steps, and core research findings. The workflow combines multi-omics data acquisition, quantitative proteomic and PTM profiling, and bioinformatic visualization analyses to construct a comprehensive aging-related PTM regulatory network in CRC. Arrows indicate the data flow and logical connections between analytical steps

Aging is a complex and multifactorial biological process closely linked to tumour initiation, progression, and therapeutic resistance [36]. In CRC, aging-related molecular reprogramming not only remodels the tumor microenvironment but also reshapes intracellular signaling pathways through diverse PTMs. In this study, we integrated multi-PTM omics data to characterize the phosphorylation, malonylation, and ubiquitination landscapes in CRC, revealing how their interplay coordinates distinct molecular events that drive cellular senescence. Our results demonstrate that these PTMs exhibit high heterogeneity and dynamic regulation during CRC cell senescence, participating in multi-layered biological processes ranging from signal sensing to transcriptional control, thereby constituting a critical molecular foundation of the “aging–cancer axis” (Fig. 7).

Our subcellular localization analysis revealed distinct spatial distributions of differentially modified proteins: phosphorylated proteins predominantly localize in the cytoplasm [19], malonylated proteins are enriched in mitochondria [37], and ubiquitinated proteins display broader distributions, including those in the cytoplasm, nucleus, plasma membrane, and peroxisomes [38]. This coupling between modification types and subcellular compartments reflects the spatial division of labor and coordination among PTMs in orchestrating senescence processes. Importantly, such spatial specificity not only clarifies the regulatory hierarchy of modifications within key aging pathways but also offers critical clues for dissecting PTM-mediated aging mechanisms from an organelle-specific perspective.

We further identified significant differences in expression and functional enrichment among the three PTM classes. The Phosphorylation-differential proteins are associated mainly with signal transduction and cell cycle regulation, the ubiquitination-differential proteins are related to protein degradation, the stress response, and genomic stability, whereas the malonylation-differential proteins are closely associated with mitochondrial metabolism and energy homeostasis. These findings suggest that CRC cells adapt to senescence-induced multifactorial stresses by differentially activating multiple PTM pathways, demonstrating remarkable adaptability to microenvironmental changes.

Moreover, LMNB1 was found to harbor three differential PTMs across three functional domains, suggesting that these sites may cooperatively regulate chromatin architecture, nuclear scaffold dynamics, and DNA damage response pathways to modulate cell fate decisions during senescence [39]. The convergence of spatial enrichment and functional localization supports the notion that multisite modifications synergistically control specific protein functions to drive cellular state transitions. In terms of clinical applications, the identification of aging-related PTMs as regulatory hubs in CRC holds significant translational potential. Due to their dynamic and reversible nature, PTMs can sensitively reflect cellular states such as stress, senescence, and malignant transformation, providing more precise and real-time indicators than conventional genomic or transcriptomic biomarkers [40]. The discovery of LMNB1 multi-site modifications and other PTM-regulated proteins highlights potential targets for diagnosis and therapy. Future efforts should focus on developing clinically applicable assays, such as targeted mass spectrometry or PTM-specific antibodies, and validating their diagnostic and prognostic value through prospective clinical studies.

Our proteomic analysis revealed decreased phosphorylation of EGFR at T693 and S991, together with reduced ubiquitination at K716 and K970, two of which are novel sites located within the C-terminal regulatory region that mediates receptor endocytosis and degradation. Phosphorylation at T693 or adjacent residues has been reported to create docking motifs for CBL-family E3 ligases, which catalyze K63-linked ubiquitination and promote EGFR internalization and lysosomal degradation [30, 41]. Therefore, the concomitant reduction in phosphorylation and ubiquitination observed in our dataset may reflect impaired E3 ligase recruitment, leading to delayed receptor internalization and increased EGFR stability. Given that EGFR overexpression and stabilization are frequently observed in CRC tissues [42], such post-translational changes could sustain MAPK and PI3K–AKT signaling, thereby promoting tumor cell proliferation and survival [43]. In contrast, we identified increased ubiquitination of ATM at K1317 and K2148 (both novel sites), accompanied by enhanced phosphorylation of TP53BP1 at S1068, a well-established marker of ATM activation during the DNA damage response [44]. Non-degradative K63-linked ubiquitination has been shown to facilitate ATM activation through E3 ligases such as RNF8 and RNF168 [45, 46]; thus, the elevated ubiquitination observed here may represent a regulatory modification that enhances ATM-mediated phosphorylation of p53 (S15) and induction of p21, contributing to senescence-associated growth arrest. Collectively, these findings suggest that the newly identified phosphorylation and ubiquitination sites on EGFR and ATM act as modulatory elements that fine-tune receptor turnover and DNA damage response signaling, providing mechanistic insight into how aging-related PTM remodeling may reshape EGFR-driven proliferative signaling and ATM–p53–p21-mediated senescence pathways in CRC.

Based on these mechanistic insights, combinatorial therapeutic approaches targeting multiple PTMs, such as co-modulating EGFR phosphorylation/ubiquitination and ATM ubiquitination/kinase activity may help restore signaling balance and improve treatment efficacy in aging-related CRC [47–49]. Research indicates that PTMs are not isolated events, but rather part of a complex network of PTM crosstalk [50]. Although our study did not directly test therapeutic interventions, the coexistence of multiple PTMs on key signaling proteins suggests potential for combinatorial targeting [51]. We propose that modulating distinct PTMs simultaneously may yield synergistic antitumor effects, a hypothesis to be evaluated in future CRC cell and organoid models.

Despite these insights, our study has several limitations. The relatively small sample size necessitates further validation with expanded cohorts. The study predominantly relies on a single-center sample source, underscoring the need for multicenter verification. Additionally, while our multi-omics analysis revealed potential regulatory roles of novel PTM sites, the causal relationships between these modifications and aging phenotypes in CRC remain to be experimentally established.

Building on our current findings, future work will focus on several key challenges in applying this approach to larger and more diverse CRC patient groups. Technically, it will be important to develop reliable methods to detect and measure PTMs across different sample types and research centers. Biologically, variations among patients and tumor heterogeneity may make it difficult to identify consistent aging-related PTM patterns. To address these issues, we plan to carry out multicenter studies using standardized procedures and perform time-course analyses in organoid, patient-derived xenograft (PDX), and clinical models to better understand how PTM changes evolve during tumor aging and progression.

## Conclusion

By integrating phosphorylation, malonylation, and ubiquitination, this study systematically constructed a comprehensive network map of aging regulation in CRC, revealing how PTMs collaboratively modulate key molecular pathways involved in cancer-associated senescence. These findings highlight the critical role of PTMs of proteins such as LMNB1 in regulating cellular senescence and tumor microenvironment adaptability, providing novel theoretical foundations for aging-targeted interventions and biomarker development in CRC.

## Data Availability

The mass spectrometry proteomics data have been unloaded and deposited onto the ProteomeXchange Consortium through the PRIDE partner repository: PXD028504, PXD021314, PXD021318. The gene set associated with mitochondrial dysfunction was referenced from the HPA database (The Human Protein Atlas).

## Abbreviations

CRC: Colorectal cancer
PTMs: Post translational modifications
PPI: Protein–protein interaction
GO: Gene Ontology
KEGG: Kyoto Encyclopedia of Genes and Genomes
FBXW7: F-box and WD repeat domain-containing 7
EGFR: Epidermal Growth Factor Receptor
ATM: Ataxia Telangiectasia Mutated
TP53BP1: Tumor Protein p53 Binding Protein 1
HSPD1: Heat Shock Protein Family D Member 1
ROS: Reactive oxygen species
mtUPR: Mitochondrial unfolded protein response
FDR: False discovery rate
DDR: DNA damage response
PSMs: Peptide-spectrum matches
PDX: Patient-derived xenograft
